# Chronic Rhinosinusitis Patients Show Accumulation of Genetic Variants in *PARS2*

**DOI:** 10.1371/journal.pone.0158202

**Published:** 2016-06-27

**Authors:** Viktor Henmyr, Christina Lind-Halldén, Christer Halldén, Torbjörn Säll, Daniel Carlberg, Claus Bachert, Lars-Olaf Cardell

**Affiliations:** 1 Biomedicine, Kristianstad University, Kristianstad, Sweden; 2 Department of Biology, Lund University, Lund, Sweden; 3 Upper Airways Research Laboratory, University Hospital Ghent, Ghent, Belgium; 4 Division of ENT Diseases, CLINTEC, Karolinska Institutet, Huddinge, Sweden; Westmead Institute for Medical Research, AUSTRALIA

## Abstract

Genetic studies of chronic rhinosinusitis (CRS) have identified a total of 53 CRS-associated SNPs that were subsequently evaluated for their reproducibility in a recent study. The rs2873551 SNP in linkage disequilibrium with *PARS2* showed the strongest association signal. The present study aims to comprehensively screen for rare variants in *PARS2* and evaluate for accumulation of such variants in CRS-patients. Sanger sequencing and long-range PCR were used to screen for rare variants in the putative promoter region and coding sequence of 310 CRS-patients and a total of 21 variants were detected. The mutation spectrum was then compared with data from European populations of the 1000Genomes project (EUR) and the Exome Aggregation Consortium (ExAC). The CRS population showed a significant surplus of low-frequency variants compared with ExAC data. Haplotype analysis of the region showed a significant excess of rare haplotypes in the CRS population compared to the EUR population. Two missense mutations were also genotyped in the 310 CRS patients and 372 CRS-negative controls, but no associations with the disease were found. This is the first re-sequencing study in CRS research and also the first study to show an association of rare variants with the disease.

## Introduction

Chronic rhinosinusitis (CRS) is a disorder of the nasal cavity and paranasal sinuses characterized by persistent inflammation. Based on the presence or absence of nasal polyps, the disease can be divided into CRS with nasal polyps (CRSwNP) and CRS without nasal polyps (CRSsNP). The mechanisms of the disease are largely unknown, but it is believed to result from a combination of environmental factors and the genetic background of affected individuals [1]. The genetics of CRS has been thoroughly reviewed with respect to the involvement of different genes and molecular mechanisms [2]. Previous genetic studies primarily focused on candidate genes involved in inflammatory response and innate immunity and many were based on limited population sizes. Together these studies defined a set of 53 CRS-associated single nucleotide polymorphisms (SNPs) that were evaluated for reproducibility in a recent study [3]. This test for CRS associations in 613 cases and 1588 background population controls revealed a total of 7 SNPs with *P* values < 0.05. The rs2873551 SNP, in linkage disequilibrium (LD) with the prolyl-tRNA synthetase 2 (*PARS2*, ENSG00000162396) gene had the lowest *P*-value and was significantly associated with CRS also after a Bonferroni correction (*P* = 0.00022; *Q* = 0.0054; OR = 0.77). This SNP has earlier been associated with CRS in a Canadian pooled-GWAS of 173 cases and 130 controls (*P* = 0.00003; OR = 0.5) [4] and was thus initially identified in a hypothesis-free study. *PARS2* is located in a 7.62 kbp region on chromosome 1p32 and consists of two exons where only one is protein coding. The gene encodes an enzyme consisting of 475 amino acids that is imported to the mitochondrion where it charges proline to tRNA molecules. Since *PARS2* showed the strongest association in the previous replication study, the present study aims to comprehensively screen for genetic variants in the *PARS2* gene by long-range PCR (LR-PCR) and re-sequence analysis. This is the first re-sequencing study and also the first study to evaluate the contribution of low-frequency and rare variants in CRS disease. Functional assessment of the detected variants will be made using comparison with sequence data from the 1000Genomes project and the Exome Aggregation Consortium (ExAC) and by functional prediction analysis.

## Material and Methods

### Ethics statement

The study was carried out after approval by the Ethics Committee of Ghent University Hospital, Belgium and the Ethics Committee of the Medical Faculty, Lund University, Sweden, and written informed consent was obtained from each participant before inclusion in the study.

### Study population and disease definitions

Whole blood samples were obtained from 310 chronic rhinosinusitis patients (195 male and 115 female patients, mean age 46) who underwent routine surgical intervention unrelated to the present study for the treatment of CRS with (n = 138) and without (n = 172) nasal polyps at the Ear, Nose, and Throat Department, University Hospital, Ghent, Belgium. The diagnosis of sinus disease was based on medical history, clinical examination, nasal endoscopy, and computed tomographic scanning of the sinuses [3]. The atopic status of all patients was evaluated by skin prick tests to the most frequent inhalant allergens [5]. The 372 CRS negative controls were recruited at Malmö University Hospital, Sweden, in 2003–2009 and consist of unrelated subjects from the general population. Controls had no history of CRS or any other atopic diseases and had a negative skin prick test or Phadiatop test [6]. Genomic DNA was extracted from blood collected in EDTA using QIAamp 96 DNA Blood kit (Qiagen, Hilden, Germany) and DNA concentrations were determined by fluorometry using PicoGreen (Molecular Probes, Invitrogen, Eugene, OR, USA).

### Long range-PCR

LR-PCR systems were designed using NCBI/Primer-BLAST (http://www.ncbi.nlm.nih.gov/tools/primer-blast/; S1 Table). PCR was performed in a total reaction volume of 5 μl, containing 10 ng of template DNA, 0.1 U KAPAExtra Hot Start PCR enzyme mix (Kapa Biosystems Ltd, Cape Town, South Africa), 1X LongRange PCR buffer, 0.3 mM dNTPs and 0.4 μM of each primer. The PCR products were visualized on 1% agarose gels and analyzed with the Image Lab Software version 3.0 (Bio-Rad Laboratories Inc., Hercules, CA, USA).

### Sanger sequencing

All DNA sequences used for primer design were obtained from the GRCh37 assembly through the Ensembl Genome Browser (http://www.ensembl.org). Primers were designed using Primer-BLAST and purchased from DNA Technology A/S (Risskov, Denmark; S1 Table). Big Dye Terminator Sanger sequencing was performed in both directions using a 3130XL Genetic Analyzer (Life Technologies, Carlsbad, CA, USA). Primary PCR was performed using KAPA Taq Extra HS PCR Kit (KAPA Biosystems) and primary PCR products were treated with ExoSAP-IT^®^ (Life Technologies) according to the manufacturer’s instructions. DNA sequencing was subsequently performed in a total volume of 5 μl containing 0.5X Big Dye sequencing ready reaction premix (Big Dye Terminator v 2.0, Life Technologies), 0.5X Big Dye Sequencing buffer and 3.2 pmol of the sequencing primer. The sequencing reactions were purified using Xterminator (Life Technologies) according to the manufacturer’s instructions. Sequences were interpreted and all polymorphisms were identified using SeqScape ver. 2.5 (Life Technologies) and confirmed by manual inspection.

### Genotyping

TaqMan assay design was made through the assay design pipeline of Life Technologies for rs35201073 and rs11577368 and TaqMan genotyping was performed according to the manufacturer’s instructions on a CFX384 PCR machine (Bio-Rad Laboratories Inc.).

### Statistical analysis and bioinformatics

Statistical analyses were made using R statistical software [7] and PLINK v1.07 [8]. Genotype frequencies were calculated and tested for Hardy-Weinberg equilibrium. Allele frequencies were investigated for association with CRS using an ordinary χ^2^-homogeneity test. Polymorphisms were extracted from the 1000Genomes (http://www.1000genomes.org/) database. The variants were obtained from the Integrated Variant Set of the 1000Genomes Project (http://ftp.1000genomes.ebi.ac.uk/vol1/ftp/release/20110521/) release April 2012. Variants for the promoter and coding region of *PARS2* were extracted for 1092 individuals using tabix [9]. The promoter region was defined according to the ~1000bp core promoter element annotated at the Ensembl genome browser (ENSR00000165397). The average coverage of the sequence data was > 50X in the coding regions and 2-6X in the promoter regions [10]. Using VCFtools [11] this data set was then subdivided into four separate populations; individuals of European (EUR; 379 individuals), African (AFR; 246), Asian (ASN; 286) and South American origin (AMR; 181). The EUR population consists of five different subpopulations: 89 individuals from England and Scotland (GBR), 85 from Utah with northern and western European ancestry (CEU), 98 from Italy (TSI), 93 from Finland (FIN) and 14 from Spain (IBS). Allele frequencies were calculated for each variation using the same allele as referent for all populations. Missense mutations identified in the study populations were investigated using SIFT and PolyPhen2 [12, 13].

### Permutation tests and simulations

To test for accumulation of rare (MAF <1%) and low-frequency (MAF <5%) variants in the coding and promoter regions compared to the 1000Genomes EUR population a permutation test was made. In this test, rare variants from both the CRS patients and the EUR population were pooled. Each variant were then randomly assigned to either population. A score was tallied each time the CRS population had an equal or greater number of alleles than the observed number. This was repeated 100 000 times. The test quantity was calculated as the total score over the number of iterations. A similar test was performed for accumulation of haplotypes where the total count of individual haplotypes of the CRS patients and the EUR population were pooled.

A test for any accumulation of variants in the coding region of *PARS2* was also made using data from ExAC (Cambridge, MA (URL: http://exac.broadinstitute.org) [date accessed (02, 2015)]) [14]. ExAC is a resource containing allele frequencies from 60 706 unrelated individuals from various disease-specific and population genetic studies. The non-Finnish European population of ExAC consists of > 30 000 individuals. A total of 139 SNVs (excluding indels) present in the coding region of *PARS2* were extracted from ExAC for this population. For each of the 100 000 iterations, the number of expected variants were calculated using the minor allele frequency from ExAC data as the probability of finding a variant for 620 chromosomes, corresponding to the number of chromosomes in the CRS population. The number of variants from the simulation test were then compared with the number of detected variants in the 310 CRS patients for three different categories; 1) total number of alternative allele counts of variants with any MAF, 2) total number of alternative allele counts of low-frequency variants (MAF <5%) and 3) total number of alternative allele counts of rare variants (MAF <1%). A score was tallied each time the simulated dataset from ExAC had an excess of variants compared to the CRS patients. The test quantity was calculated as the total score over the number of iterations.

### Haplotype reconstruction

Using data from the EUR population (379 individuals) Haploview [15] was used to produce a linkage disequilibrium map over the *PARS2* gene region including 15 kbp flanking regions (S1 Fig). PHASE v.2.1.1 was used to estimate haplotypes for the CRS population based on the 4 most common SNPs in the region and the previously identified SNP associated with CRS [3]. To construct a genealogy of the haplotypes, the Chimpanzee allele information from dbSNP was investigated for the five positions in order to identify an ancestral haplotype. The African population from the 1000Genomes project was then investigated with respect to the haplotypes for these five SNPs. The following principles have been used in the construction of the genealogy: 1) we assume that rare haplotypes tend to be younger than more common haplotypes 2) when recombination is inferred it is the rarer haplotype that is the offspring rather than vice versa 3) if there is a possibility that several different parental haplotypes has produced the same recombinant haplotype it is more likely that the true parental haplotypes are common rather than rare. Using these principles it was possible to construct a hypothetical genealogy.

## Results and Discussion

Two overlapping LR-PCR systems covering the *PARS2* gene region were used to screen for insertions and deletions in 310 CRS patients. Gel electrophoresis of the LR-PCR fragments yielded clear and sharp bands, but no abnormal band pattern could be detected. Thus, no insertions or deletions were found. The coding sequence and approximatly1000 bp of the putative promoter region of *PARS2* were then re-sequenced in the 310 CRS patients to screen for rare variants. In the promoter region a total of 10 variants were detected (Table 1). Four of these had minor allele frequencies (MAF) ≥ 1% and 6 were present on single chromosomes. In the coding sequence a total of 11 variants were detected. Only 4 of these had MAF ≥ 1% whereas the remaining 7 were rare and only present on one or two chromosomes each. A total of 7 variants were missense mutations and 2 of those were classified as damaging by SIFT.

**Table 1:** Promoter and coding variants found in 310 CRS patients.

			MAF				
Position*	SNP ID	Alleles^†^	CRS	Public db^∞^	AA-change	SIFT^¥^	PolyPhen2^§^
55223677	rs143717155	**G**/A	0.0016	0.0011	Synonymous		
55223744	rs35201073	**G**/C	0.0032	0.0015	P364R	DAM	Prob
55223859	NEW	**T**/C	0.0016		T326A	TOL	Ben
55223908	rs370234936	**G**/A	0.0016	0.0000030	Synonymous		
55223992	rs145005088	**G**/A	0.011	0.0065	Synonymous		
55224120	NEW	**G**/C	0.0016		L239V	TOL	Ben
55224131	rs2270004	**T**/C	0.17	0.16	N235S	TOL	Ben
55224580	rs145866387	**G**/A	0.0032	0.000060	Synonymous		
55224672	rs74617964	**G**/A	0.0016	0.000060	R55W	TOL	Pos
55224751	rs11577368	**C**/A	0.18	0.16	R28S	DAM	Ben
55224773	rs116816976	**A**/C	0.026	0.021	L21R	TOL	Ben
55229346	rs768053281	**A**/C	0.0016		Promoter		
55229354	rs1180947	**A**/G	0.048	0.057	Promoter		
55229483	NEW	**C**/A	0.0016		Promoter		
55229523	rs1180946	C/**G**	0.44	0.45	Promoter		
55229527	rs61768813	**C**/T	0.0016	0.0013	Promoter		
55229576	NEW	**T**/C	0.0016		Promoter		
55229835	rs12023572	**C**/T	0.15	0.17	Promoter		
55229864	rs563439229	**C**/T	0.0016		Promoter		
55230227	rs116416055	**G**/A	0.0016	0.0013	Promoter		
55230233	rs1180945	T/**C**	0.44	0.45	Promoter		

* Position on chromosome 1 according to GRCh37

^†^ Major alleles are given in bold

^¥^ SIFT: TOL; TOLERATED, DAM; DAMAGING.

^§^ PolyPhen2: Ben; Benign, Pos; Possibly Damaging, Prob; Probably Damaging

^∞^ Minor allele frequencies was collected from ExAC for coding regions and 1000Genomes for promoter regions

**Fig 1:**
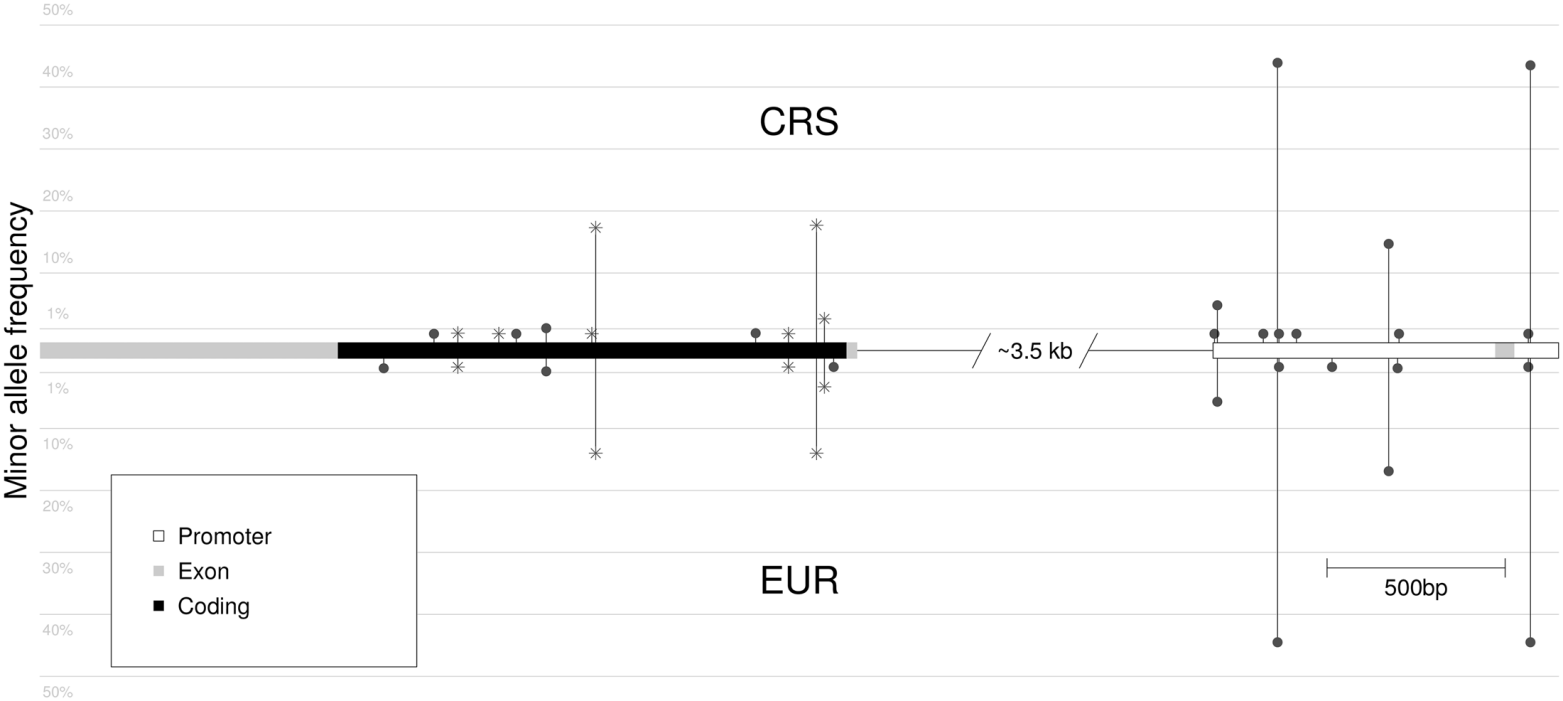
Comparison of variants found in 310 CRS patients and 379 EUR background population controls from the 1000Genomes project. Circles denote synonymous mutations and stars denote missense mutations. Intron 1 has been shortened by 3.5 kb to increase resolution.

The mutation spectrum of the CRS patients was compared with that obtained from the 379 EUR individuals, showing that all variants present in either population at a MAF ≥ 1% were present also in the other population (Fig 1 and S2 Table). Contrary to this pattern, a majority of the variants with MAF < 1% were unique to either population; 4 were unique to the EUR population, 9 were unique to the CRS population whereas 4 were in common to both populations. Thus, there is a surplus of rare variants in the CRS patients compared with the EUR population in the investigated region. The significance of the accumulation of variants with MAF < 1% was evaluated using a one-sided permutation test yielding a non-significant but relatively low *P*-value for the coding sequence (*P*_*coding*_ = 0.070, *P*_*promoter*_ = 0.36).

Further evaluation of the coding region was made using data from > 30 000 non-Finnish Europeans extracted from the ExAC database. A total of 137 variants were identified and a simulation was performed to test for an accumulation of both rare and low-frequency variants, as well as variants with any MAF in the CRS population compared to the ExAC population. A significant surplus of variation was observed in the CRS population (*P* = 0.048). This effect was enhanced for alleles with MAF < 5% (*P* = 0.039), but was non-significant for alleles with MAF < 1% (*P* = 0.12). No significances were detected when subdividing the CRS population into CRS with and without nasal polyps. Altogether, the results above indicate an overall accumulation of variants in the coding sequence of *PARS2* in CRS patients.

The present investigation shows an accumulation of low-frequency variants in CRS patients compared with the 1000Genomes and ExAC populations. Although rare variants did not reach significance in the present study, both common (rs2873551 SNP [3]) and low-frequency variants of the *PARS2* locus have now been significantly associated with the CRS phenotype. However, the functional role of *PARS2* in CRS is difficult to understand. Little is known about this gene and except for its association with CRS, it has only been associated with one additional disease. Variants in a compound heterozygote state have previously been identified in a patient with Alpers syndrome, which is a progressive neurodegenerative disorder [16]. In general, diseases caused by mutations in mitochondrial aminoacyl-tRNA synthetases (mtARS) often affect the brain and results in severe phenotypes. Since mtARS have such a fundamental role in protein biosynthesis, a complete loss of function of the respective enzymes is not compatible with life [17]. Thus, there are good reasons to expect strong purifying selection acting on *PARS2*. Two predictions can therefore be made, the first is that the scaled number of variable sites should be larger than the average number of pairwise differences (PI). This is the case for both the CRS (0.68 vs 1.71) and the EUR (0.57 vs 1.21) populations. The second is a low overall level of variation. The standard measure is PI per bp (pi) which is 0.0005 and 0.0004 respectively which is clearly lower than the average of 0.0008 [18]. In the ExAC population the same low level of variability is seen (0.0004). The difference between K/a and PI is extreme, 0.622 vs 12.3. As a matter of fact, only two of the variants have a frequency > 10%, one has a frequency of 2% and the remaining variants are below 1%. The overall pattern is thus fully compatible with selection acting to conserve the sequence and thus the function of *PARS2*.

In addition to the search for rearrangements and rare variants a standard association analysis was made for two SNPs. A total of 310 CRS patients and 372 CRS-negative controls were subsequently genotyped for the missense variants that were predicted to be damaging according to SIFT: rs35201073 and rs11577368 (rs2270004). The rs11577368 SNP showed a higher MAF in the CRS-negative controls (20%) compared to the CRS patients (17%) in contrast to what was observed in the EUR population (14%). No frequency difference was observed for the rs35201073 SNP (0.3%). Thus, the missense variants predicted to be damaging show no obvious association to CRS, indicating that it may be alterations affecting expression rather than changes in the amino acid sequence that is contributing to the CRS phenotype.

**Fig 2:**
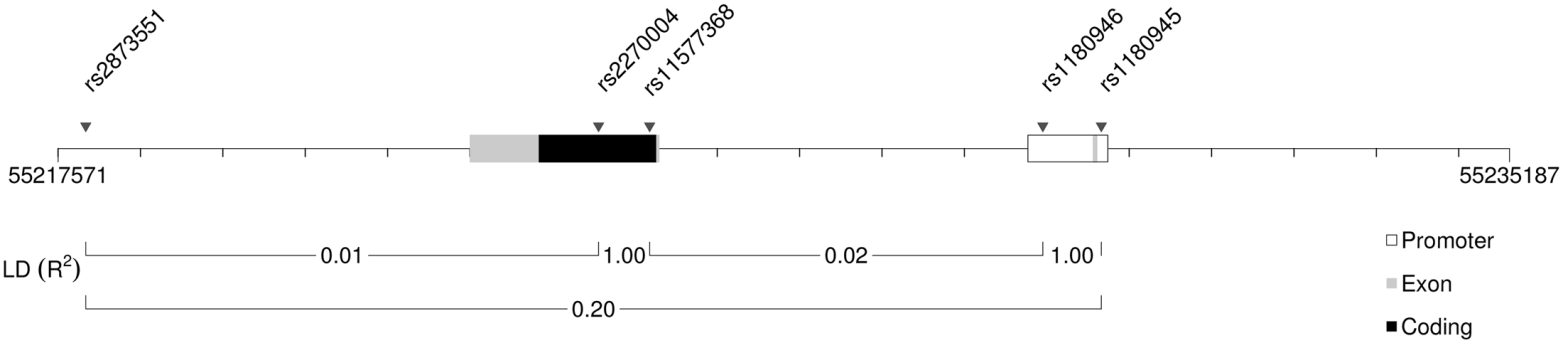
*PARS2* gene region with exons and analyzed SNP markers. The linkage disequilibrium pattern (LD) are reported as R^2^ and estimated using the EUR population of the 1000Genomes project.

PHASE was used to estimate haplotypes for the CRS population based on the 4 most common SNPs in the region and the previously identified SNP associated with CRS [3] (Fig 2). All 5 SNPs are located within the same haploblock and LD within this block is rather low when LD is quantified as R^2^ but high when measured as D´ (S1 Fig). This is a direct consequence of the pattern of genetic variation with a relatively large number of rare variants. A site with a rare allele will almost always show D´ = 1 with other sites/loci but will never show R^2^ = 1. Seven different haplotypes were identified in the CRS patients (H1–H7). Four of these had a frequency ≥ 5% (Table 2). The corresponding haplotypes were extracted from 1000Genomes project data for the 379 EUR individuals. In the EUR population only four different haplotypes were found. These are identical to the four common haplotypes of the CRS population. A permutation test showed that the surplus of the 3 rare haplotypes (H5–H7) of the CRS population was significant (*P* = 0.0044).

**Table 2:** Haplotype frequencies estimated for the CRS population compared to the haplotype frequencies of the EUR population.

ID	rs2873551	rs2270004	rs11577368	rs1180946	rs1180945	Frequency	
						CRS	EUR*
H1	T	T	C	C	T	0.43	0.44
H2	C	T	C	G	C	0.33	0.36
H3	T	C	A	G	C	0.17	0.14
H4	T	T	C	G	C	0.050	0.050
H5	T	T	A	G	C	0.0048	-
H6	T	T	C	C	C	0.0048	-
H7	T	C	A	G	T	0.0016	-

* Haplotype frequencies obtained from the Integrated Variant Set of the 1000Genomes Project (http://ftp.1000genomes.ebi.ac.uk/vol1/ftp/release/20110521/) release April 2012.

The haplotypes from the African part of the 1000Genomes project (AFR), as well as the chimpanzee haplotype for these 5 positions were extracted from the "Ancestral allele" field in dbSNP. Nine different haplotypes were found in the AFR population, and these formed together with the CRS haplotypes 11 different haplotypes. One of these haplotypes were identical to the chimpanzee haplotype. Assuming that this is the ancestral haplotype and that no recurrent mutation has occurred, a genealogy for the haplotypes observed in the CRS patients and the African population was reconstructed (S2 Fig). The African haplotypes (A1–A9) are assumed to have arisen through either one or two mutational steps from the ancestral haplotype (H4/A3) in combination with recombination between common haplotypes in certain cases. Five of the 7 haplotypes of the CRS population are present also in the African population (H1–H4, H6). In general, the more common European haplotypes are also common in the African population, e.g. H1/A1, H2/A2, H3/A5 and H4/A3. Certain haplotypes have arisen through population-specific recombination events, e.g. H5, H7, A7–A9. The ancestral haplotype is the third most common in AFR. All the remaining of the six most common haplotypes in AFR is one or two mutational steps from the ancestor and together represents all 5 SNPs. In the CRS population all of the 9 CRS-specific variants are located on any of the four common haplotypes and not on the three rare ones (S3 Table). This is compatible with an expected accumulation of rare variants on the common haplotypes according to their population frequencies.

The *PARS2* gene produced a significant association signal in a recent replication study of all known CRS-associations and is now the first human gene to be re-sequenced within CRS research. *PARS2* showed an accumulation of low-frequency variants in CRS patients compared to background populations. However, the functional role of *PARS2* in CRS is presently unknown and even if variants in *PARS2* are truly associated with CRS, the clinical relevance would be limited as the variation would only explain a small proportion of the disease population. Given the observed association with CRS for both common and low-frequency *PARS2* variants, further studies of the involvement of this gene in CRS is warranted. Obvious studies include replication in additional populations and expression studies in CRS patients versus controls in relevant tissues.

## Supporting Information

S1 Fig Linkage disequilibrium plot of *PARS2*. (PDF)

S2 Fig Haplotype genealogy of *PARS2*. (PDF)

S1 Table Primers used for LR-PCR and Sanger sequence analysis of *PARS2*. (DOCX)

S2 Table Comparison of promoter and coding variants found in 310 CRS patients and 379 European background population individuals from the 1000Genomes project. (DOCX)

S3 Table Distribution of rare CRS-specific variants on *PARS2* haplotypes H1–H7. (DOCX)
