# Supplementary figures and images for: Chronic Rhinosinusitis Patients Show Accumulation of Genetic Variants in PARS2
Source: PLoS One. 2016 Jun 27;11(6):e0158202. doi: 10.1371/journal.pone.0158202 (PMC4922623; doi:10.1371/journal.pone.0158202)

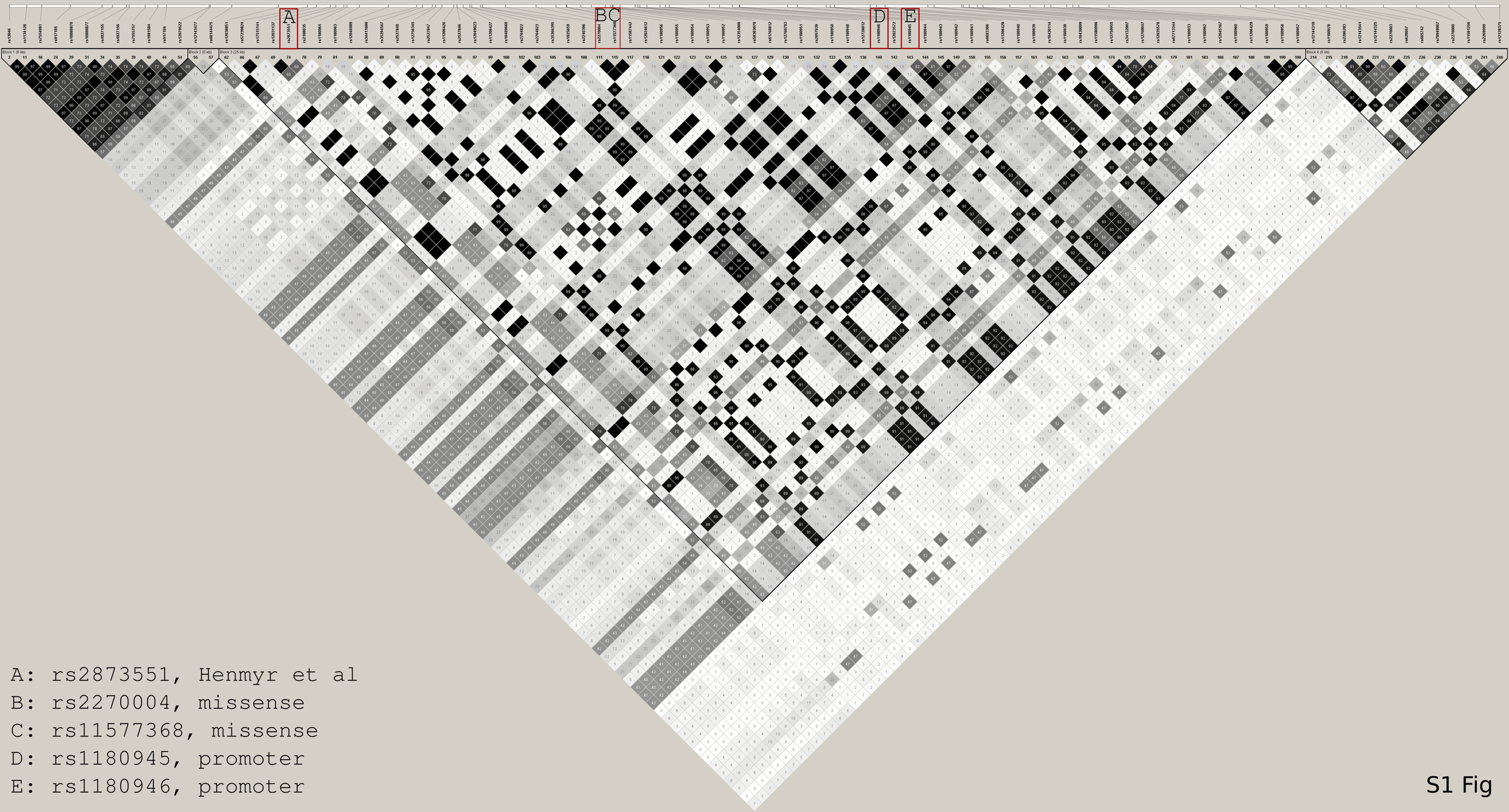

A: rs2873551, Henmyr et al  
B: rs2270004, missense  
C: rs11577368, missense  
D: rs1180945, promoter  
E: rs1180946, promoter

S1 Fig

Supplement: S1 Fig — (PDF) [file pone.0158202.s001.pdf]

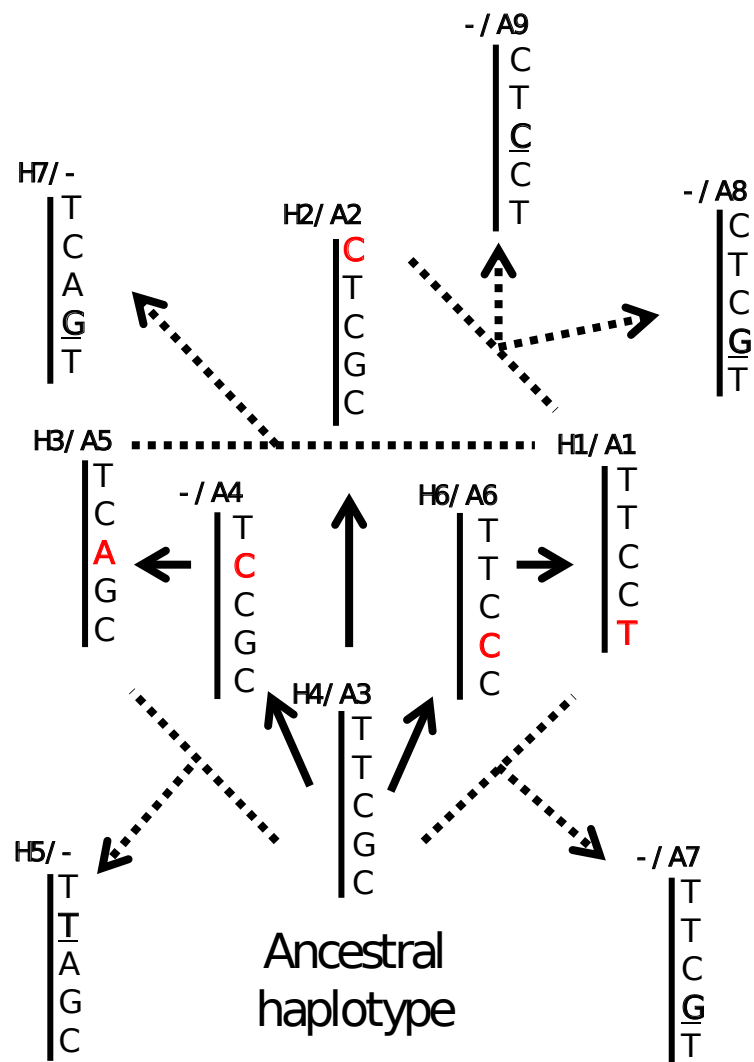

Supplement: S2 Fig — (PDF) [file pone.0158202.s002.pdf]
