# Supplementary material for: Chronic Rhinosinusitis Patients Show Accumulation of Genetic Variants in PARS2
Source: PLoS One. 2016 Jun 27;11(6):e0158202. doi: 10.1371/journal.pone.0158202 (PMC4922623; doi:10.1371/journal.pone.0158202)
Supplement: S1 Table — (DOCX) [file pone.0158202.s003.docx]

| **S1 Table**. Primers used in long-range PCR and Sanger sequence analysis. | | | | |
| --- | --- | --- | --- | --- |
| Primer ID | Position^*^ | Sequence | Amplicon size (bp) | Annealing temp (°C) |
| Sanger_F1 | 55228965-55228985 | 5'-GGCATTGGAAGCGAATGGTG-3' | 774 | 60 |
| Sanger_R1 | 55229719-55229739 | 5'-GCCTAGGGTTCCATTGCTGT-3' |  |  |
| Sanger_F2 | 55229619-55229638 | 5'-TGTGTCAAGCCACACAGCA-3' | 755 | 60 |
| Sanger_R2 | 55230354-55230374 | 5'-CAACGATCTTGGGGAAAGCC-3' |  |  |
| Sanger_F3 | 55222400-55222420 | 5'-CCCGATACTTGGGGGAAGAA-3' | 752 | 57 |
| Sanger_R3 | 55223132-55223152 | 5'-TAGGAGGCTGAGAAGCAGGA-3' |  |  |
| Sanger_F4 | 55223032-55223053 | 5'-TCAGCCATCAGCCATTCTCAT-3' | 805 | 57 |
| Sanger_R4 | 55223817-55223837 | 5'-CTGGCTGAAATGGGGTGCTA-3' |  |  |
| Sanger_F5 | 55223712-55223732 | 5'-ATGAGGCAGGCTTGGTAAGG-3' | 830 | 57 |
| Sanger_R5 | 55224522-55224542 | 5'-GAGAAGCTCGTGCGAGTGAT-3' |  |  |
| Sanger_F6 | 55224418-55224438 | 5'-CTTTGCCCATCAAGTCCCAC-3' | 632 | 57 |
| Sanger_R6 | 55225025-55225050 | 5'-TCAAAAACGGAAGTAAGAGGTTAGA-3' |  |  |
| LR-PCR_F1 | 55220581-55220601 | 5'-CACTAGCCCAAAAGGGAGGG-3' | 7051 | 60 |
| LR-PCR_R1 | 55227613-55227632 | 5'-TTTCTGGCTGGAGCCACTG-3' |  |  |
| LR-PCR_F2 | 55224951-55224971 | 5'-TCGAAGGCAGCCACTGATTT-3' | 7502 | 60 |
| LR-PCR_R2 | 55232433-55232453 | 5'-TCCAGGATCGGAAGGGATGT-3' |  |  |
| ^*^ According to GRCh37 | |  |  |  |
