# Supplementary material for: Chronic Rhinosinusitis Patients Show Accumulation of Genetic Variants in PARS2
Source: PLoS One. 2016 Jun 27;11(6):e0158202. doi: 10.1371/journal.pone.0158202 (PMC4922623; doi:10.1371/journal.pone.0158202)
Supplement: S2 Table — (DOCX) [file pone.0158202.s004.docx]

| **S2 Table.** Comparison of promoter and coding variants found in 310 CRS patients and 379 European background population individuals from the 1000Genomes project. | | | | | | | |
| --- | --- | --- | --- | --- | --- | --- | --- |
| **Variant information** | | |  | **MAF** | |  |  |
| **Position** | **SNP ID** | **Alleles** |  | **CRS** | **EUR** | **AA-change** | **SIFT** |
| 1:55223536 | rs141040125 | **C**/T |  |  | 0.003 | Synonymous |  |
| 1:55223677 | rs143717155 | **G**/A |  | 0.002 |  | Synonymous |  |
| 1:55223744 | rs35201073 | **G**/C |  | 0.003 | 0.001 | P364R | DAMAGING |
| 1:55223859 | pos_55223859 | **T**/C |  | 0.002 |  | T326A | TOLERATED |
| 1:55223908 | pos_55223908 | **G**/A |  | 0.002 |  | Synonymous |  |
| 1:55223992 | rs145005088 | **G**/A |  | 0.011 | 0.008 | Synonymous |  |
| 1:55224120 | pos_55224120 | **G**/C |  | 0.002 |  | L239V | TOLERATED |
| 1:55224131 | rs2270004 | **T**/C |  | 0.173 | 0.140 | N235S | TOLERATED |
| 1:55224580 | rs145866387 | **G**/A |  | 0.003 |  | Synonymous |  |
| 1:55224672 | rs74617964 | **G**/A |  | 0.002 | 0.001 | R55W | TOLERATED |
| 1:55224751 | rs11577368 | **C**/A |  | 0.177 | 0.140 | R28S | DAMAGING |
| 1:55224773 | rs116816976 | **A**/C |  | 0.026 | 0.033 | L21R | TOLERATED |
| 1:55224799 | rs200754768 | **G**/A |  |  | 0.001 | Synonymous |  |
| 1:55229346 | pos_55229346 | **A**/C |  | 0.002 |  | Promoter |  |
| 1:55229354 | rs1180947 | **A**/G |  | 0.048 | 0.057 | Promoter |  |
| 1:55229483 | pos_55229483 | **C**/A |  | 0.002 |  | Promoter |  |
| 1:55229523 | rs1180946 | C/**G** |  | 0.439 | 0.445 | Promoter |  |
| 1:55229527 | rs61768813 | **C**/T |  | 0.002 | 0.001 | Promoter |  |
| 1:55229576 | pos_55229576 | **T**/C |  | 0.002 |  | Promoter |  |
| 1:55229676 | rs189346379 | **C**/T |  |  | 0.001 | Promoter |  |
| 1:55229835 | rs12023572 | **C**/T |  | 0.147 | 0.169 | Promoter |  |
| 1:55229860 | rs180788021 | **G**/A |  |  | 0.003 | Promoter |  |
| 1:55229864 | pos_55229864 | **C**/T |  | 0.002 |  | Promoter |  |
| 1:55230227 | rs116416055 | **G**/A |  | 0.002 | 0.001 | Promoter |  |
| 1:55230233 | rs1180945 | T/**C** |  | 0.435 | 0.445 | Promoter |  |
