# Supplementary material for: Chronic Rhinosinusitis Patients Show Accumulation of Genetic Variants in PARS2
Source: PLoS One. 2016 Jun 27;11(6):e0158202. doi: 10.1371/journal.pone.0158202 (PMC4922623; doi:10.1371/journal.pone.0158202)
Supplement: S3 Table — (DOCX) [file pone.0158202.s005.docx]

| **S3 Table.** Distribution of CRS-specific rare variants on haplotypes H1-H7. | | | | |
| --- | --- | --- | --- | --- |
| **Position** | **SNP ID** | **Number of chromosomes** | **Location** | **Haplotypes** |
| 55223677 | rs143717155 | 1 | CODING | H1,H4 |
| 55223859 | pos_55223859 | 1 | CODING | H1,H3 |
| 55223908 | pos_55223908 | 1 | CODING | H1,H3 |
| 55224120 | pos_55224120 | 1 | CODING | H1,H1 |
| 55224580 | rs145866387 | 2 | CODING | H1,H2; H2,H2 |
| 55229346 | pos_55229346 | 1 | PROM | H1,H1 |
| 55229483 | pos_55229483 | 1 | PROM | H1,H1 |
| 55229576 | pos_55229576 | 1 | PROM | H3,H3 |
| 55229864 | pos_55229864 | 1 | PROM | H3,H3 |
